# Supplementary material for: Thermodynamic Interpolation: A Generative Approach to Molecular Thermodynamics and Kinetics
Source: J Chem Theory Comput. 2025 Feb 24;21(5):2535–45. doi: 10.1021/acs.jctc.4c01557 (PMC11912209; doi:10.1021/acs.jctc.4c01557)
Supplement: Supplementary file 1 — ct4c01557_si_001.pdf [file ct4c01557_si_001.pdf]

# Supplementary Materials for:

**Thermodynamic Interpolation: A generative approach to molecular thermodynamics and kinetics**

Selma Moqvist,[†] Weilong Chen,[†] Mathias Schreiner,[†] Feliks Nüske,[‡] and Simon Olsson[*,†]

†*Department of Computer Science and Engineering, Chalmers University of Technology and University of Gothenburg, SE-41296 Gothenburg, Sweden*

‡*Max-Planck-Institute for Dynamics of Complex Technical Systems, Magdeburg, Germany*

E-mail: simonols@chalmers.se

# Supplementary Materials

## Scaling to Larger Systems

We show that the ambient TI approach, applied to MD-simulated initial conditions, displays promise in scaling to larger systems by training on a second MDQM9 molecule, consisting of 25 atoms. Due to architectural limitations, making the probability evaluation scale badly computation-wise, it is not possible to compute the Jacobian determinant for larger systems in a feasible amount of time. As such, we only produce samples from our model and no sample probabilities. Figure S1 shows the marginal histograms of torsions angles, comparing MD samples with predictions from the aTI model. Figure 6 in the manuscript shows the results of applying Time-Independent Component Analysis[1] (TICA) with lag $\tau = 2$ to the obtained torsion angles, compared to MD-simulated data.

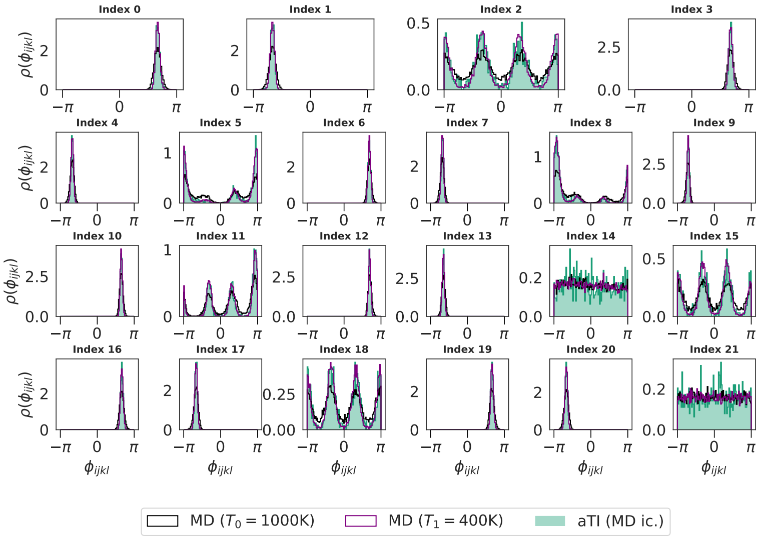


Figure S1: Marginal histograms of torsions for the 3p2y1 molecule.

## Free Energy Perturbation Methods

There exist two different estimators of the free energy $\Delta F$. One follows from the BG method,[2] which we now on denote $\Delta F^{(\mathrm{BG})}$. The second estimator follows from the TFEP

method,[3] which we will denote by $\Delta F^{(\mathrm{TFEP})}$. In the upcoming section, we derive a relationship between the two estimators and verify it experimentally using our latent TI model. An important limitation of these approaches is that the learned TI maps are never exact mappings into the target Boltzmann distribution, but rather biased versions due to modelling errors. In this section we will however assume perfect maps that do not produce errors. We denote these idealized maps as

- $f_{ZA} : \mathbf{z} \to \mathbf{x}_A$, mapping from a latent state $Z$ to the thermodynamic state $A$.
- $f_{ZB} : \mathbf{z} \to \mathbf{x}_B$, mapping from the latent state $Z$ to the thermodynamic state $B$.
- $f_{AB} : \mathbf{x}_A \to \mathbf{x}_B$, mapping directly from the initial thermodynamic state $A$ to the target thermodynamic state $B$.

In our setting, the states $A$ and $B$ are identical in all aspects but their temperatures, which are denoted $T_A$ and $T_B$ respectively.

As derived in previous work, BGs can be used to estimate free energy differences.[2] The free energy difference between two arbitrary BGs is given as the averaged difference in their loss functions. This implies that the free energy between states $A$ and $B$ can be estimated via $\Delta F^{(\mathrm{BG})}$, where the estimator is defined as

$$\Delta F^{(\mathrm{BG})} = \mathbb{E}_{\mathbf{z}}\left[\mathcal{L}_{\mathrm{KL}}^{(\mathrm{BG},\,B)}(\mathbf{z})\right] - \mathbb{E}_{\mathbf{z}}\left[\mathcal{L}_{\mathrm{KL}}^{(\mathrm{BG},\,A)}(\mathbf{z})\right], \tag{1}$$

since $\mathcal{L}_{\mathrm{KL}}^{(\mathrm{BG,X})}$ is up to a constant equal to the free energy of a single BG at state $X$. However, Equation (1) can be simplified further. Now note the definition of the Kullback-Leibler-loss

$$\mathcal{L}_{\mathrm{KL}}^{(\mathrm{BG},X)} = \mathbb{E}_{\mathbf{z}}\left[\frac{1}{kT_X}E(f_{ZX}(\mathbf{z})) - \tilde{H}_X + H_Z\right], \tag{2}$$

where $\tilde{H}_X$ is the differential entropy of the BG-generated surrogate distribution at $X$ and $H_Z$ is the differential entropy of the initial standard normal distribution. This implies that

Equation (1) can be rewritten as

$$\begin{aligned}\Delta F^{(\mathrm{BG})} &= \mathbb{E}_{\mathbf{z}}\left[E_B(f_{ZB}(\mathbf{z})) - \tilde{H}_B + H_Z\right] - \mathbb{E}_{\mathbf{z}}\left[E_A(f_{ZA}(\mathbf{z})) - \tilde{H}_A + H_Z\right] \\ &= \mathbb{E}_{\mathbf{z}}\left[E_B(f_{ZB}(\mathbf{z})) - E_A(f_{ZA}(\mathbf{z})) - \tilde{H}_B + \tilde{H}_A\right],\end{aligned} \tag{3}$$

where we for simplicity now work with reduced energies $E_A(\mathbf{x}_A) = \frac{1}{kT_A}E(\mathbf{x}_A)$ and $E_B(\mathbf{x}_B) = \frac{1}{kT_B}E(\mathbf{x}_B)$. As previously shown[2] it holds that

$$\tilde{H}_X = \mathbb{E}_{\mathbf{z}}\left[H_Z + \log|\det J_{f_{ZX}}(\mathbf{z})|\right], \tag{4}$$

which inserted into Equation (3) yields

$$\begin{aligned}\Delta F^{(\mathrm{BG})} &= \mathbb{E}_{\mathbf{z}}\left[E_B(f_{ZB}(\mathbf{z})) - E_A(f_{ZA}(\mathbf{z})) + \log|\det J_{f_{ZA}}(\mathbf{z})| - \log|\det J_{f_{ZB}}(\mathbf{z})|\right] \\ &\triangleq \mathbb{E}_{\mathbf{z}}\left[\psi(\mathbf{z})\right].\end{aligned} \tag{5}$$

As has been shown in previous work, Targeted Free Energy Perturbation (TFEP) provides a different way of estimation.[3] We denote the TFEP estimator as $\Delta F^{(\mathrm{TFEP})}$. Interestingly, this estimator is closely related to $\Delta F^{(\mathrm{BG})}$. We consider the map $\psi$, defined in the last step of Equation (5) as

$$\psi(\mathbf{z}) \triangleq E_B(f_{ZB}(\mathbf{z})) - E_A(f_{ZA}(\mathbf{z})) + \log|\det J_{f_{ZA}}(\mathbf{z})| - \log|\det J_{f_{ZB}}(\mathbf{z})|, \tag{6}$$

where $\mathbf{z} \sim \mathcal{N}(0, \mathrm{Id})$. Compared to the TFEP method, as it was originally defined,[3] we are now working with three different probability distributions:

1. The standard normal distribution $\mathcal{N}(0, \mathrm{Id})$, whose pdf we denote: $\mu_Z(\mathbf{z})$.
2. The Boltzmann distribution at temperature $T_A$, with pdf : $\mu_A(\mathbf{x}_A) \triangleq \frac{1}{\mathcal{Z}_A}\exp(-E_A(\mathbf{x}_A))$.
3. The Boltzmann distribution at temperature $T_B$, with pdf : $\mu_B(\mathbf{x}_B) \triangleq \frac{1}{\mathcal{Z}_B}\exp(-E_B(\mathbf{x}_B))$.

In total, the function $\psi$ consists of two different transformations. The first transformation

leverages the map $f_{ZA}$, mapping from a latent state $Z$ to the thermodynamic state $A$. The second transformation uses the map $f_{ZB}$ to transform from $Z$ to the second thermodynamic state $B$. Similarly to the original TFEP method,[3] the three densities are related.

- For the transformation $\mathbf{z} \to f_{ZA}(\mathbf{z}) = \mathbf{x}_A$, it holds

$$\mu_A(\mathbf{x}_A) = \frac{\mu_Z(\mathbf{z})}{|\det J_{f_{ZA}}(\mathbf{z})|}, \tag{7}$$

- For the transformation $\mathbf{z} \to f_{\mathrm{ZB}}(\mathbf{z}) = \mathbf{x}_B$, it holds

$$\mu_B(\mathbf{x}_B) = \frac{\mu_Z(\mathbf{z})}{|\det J_{f_{\mathrm{ZB}}}(\mathbf{z})|}. \tag{8}$$

Next we average over the exponentiated $\psi$ as

$$\begin{aligned}
\langle \exp(-\psi) \rangle_Z &= \int \mathrm{d}\mathbf{z} \mu_Z(\mathbf{z}) \exp(-\psi(\mathbf{z})) \\
&= \int \mathrm{d}\mathbf{z} \mu_Z(\mathbf{z}) \exp(-E_B(\mathbf{x}_B) + E_A(\mathbf{x}_A) - \log|\det J_{f_{ZA}}(\mathbf{z})| + \log|\det J_{f_{ZB}}(\mathbf{z})|) \\
&= \int \mathrm{d}\mathbf{z} \frac{\mu_Z(\mathbf{z})}{|\det J_{f_{ZA}}(\mathbf{z})|} |\det J_{f_{ZB}}(\mathbf{z})| \exp(-E_B(\mathbf{x}_B)) \exp(E_A(\mathbf{x}_A)) \\
&= \int \mathrm{d}\mathbf{z} \mu_A(\mathbf{x}_A) |\det J_{f_{ZB}}(\mathbf{z})| \exp(-E_B(\mathbf{x}_B)) \exp(E_A(\mathbf{x}_A)) \\
&= \frac{1}{\mathcal{Z}_A} \int \mathrm{d}\mathbf{z} |\det J_{f_{ZB}}(\mathbf{z})| \exp(-E_B(\mathbf{x}_B)) \\
&= \frac{1}{\mathcal{Z}_A} \int \mathrm{d}\mathbf{x}_B \exp(-E_B(\mathbf{x}_B)) = \frac{\mathcal{Z}_B}{\mathcal{Z}_A} \triangleq \exp\left(-\Delta F^{(\mathrm{TFEP})}\right).
\end{aligned} \tag{9}$$

In the last step, we invoke the definition of free energy, as written in terms of the partition functions, i.e. $F = -\log \mathcal{Z}$. Another, possibly simpler, approach to showing this is to directly write $f_{\mathrm{AB}}(\mathbf{x}_A) = f_{\mathrm{ZB}}(f_{\mathrm{ZA}}^{-1}(\mathbf{x}_A))$, implying that the direct map between $A$ and $B$ can be written in terms of the two latent maps.

Finally, we derive a relationship between $\Delta F^{(\mathrm{TFEP})}$ and $\Delta F^{(\mathrm{BG})}$ by applying the Jensen

inequality

$$f\left(\mathbb{E}\left[x\right]\right) \leq \mathbb{E}\left[f(x)\right], \tag{10}$$

which is applicable for convex functions $f$ and where $x$ is a random variable. As noted, the two estimators are given as

1. $\Delta F^{(\mathrm{TFEP})} = -\log \mathbb{E}_{\mathbf{x}_A}\left[\exp(-\varphi(\mathbf{x}_A))\right] = -\log \mathbb{E}_{\mathbf{z}}\left[\exp(-\psi(\mathbf{z}))\right]$

2. $\Delta F^{(\mathrm{BG})} = \mathbb{E}_{\mathbf{x}_A}\left[\varphi(\mathbf{x}_A)\right] = \mathbb{E}_{\mathbf{z}}\left[\psi(\mathbf{z})\right]$

We remember that $\exp(\cdot)$ is a convex function, and treat $-\varphi$ as a RV. From the Jensen inequality, we then obtain

$$\begin{aligned}
\exp\left(\mathbb{E}_{\mathbf{x}_A}\left[-\varphi(\mathbf{x}_A)\right]\right) &\leq \mathbb{E}_{\mathbf{x}_A}\left[\exp(-\varphi(\mathbf{x}_A))\right] \\
\mathbb{E}_{\mathbf{x}_A}\left[-\varphi(\mathbf{x}_A)\right] &\leq \log \mathbb{E}_{\mathbf{x}_A}\left[\exp(-\varphi(\mathbf{x}_A)\right] \\
-\mathbb{E}_{\mathbf{x}_A}\left[\varphi(\mathbf{x}_A)\right] &\leq \log \mathbb{E}_{\mathbf{x}_A}\left[\exp(-\varphi(\mathbf{x}_A)\right] \\
\Delta F^{(\mathrm{BG})} \triangleq \mathbb{E}_{\mathbf{x}_A}\left[\varphi(\mathbf{x}_A)\right] &\geq -\log \mathbb{E}_{\mathbf{x}_A}\left[\exp(-\varphi(\mathbf{x}_A)\right] \triangleq \Delta F^{(\mathrm{TFEP})}.
\end{aligned} \tag{11}$$

As can be seen from the last Equation in (11), the TFEP-estimator serves as a lower bound on the BG-estimator.

Finally, we compare the two free energy estimators and verify that the bound (11) in fact applies experimentally. Figure S2 shows the free energy evaluated with $\Delta F^{(\mathrm{BG})}$ compared to with $\Delta F^{(\mathrm{TFEP})}$, using our latent TI model to produce approximated maps $f_{ZA}^{(\theta)}$ and $f_{ZB}^{(\theta)}$. It is clear that for all target temperatures $T_B$, the estimated $\Delta F^{(\mathrm{BG})}$ is larger than the estimated $\Delta F^{(\mathrm{TFEP})}$.

## IQR-Filtering of Outliers

The estimated statistical weights $w^{l/a}$ and $\psi/\varphi$-functions in the free energy estimators can include outliers, i.e. values that empirically lie extremely far away from the remaining values. Such outliers can significantly impact the estimated quantities, especially when exponenti-

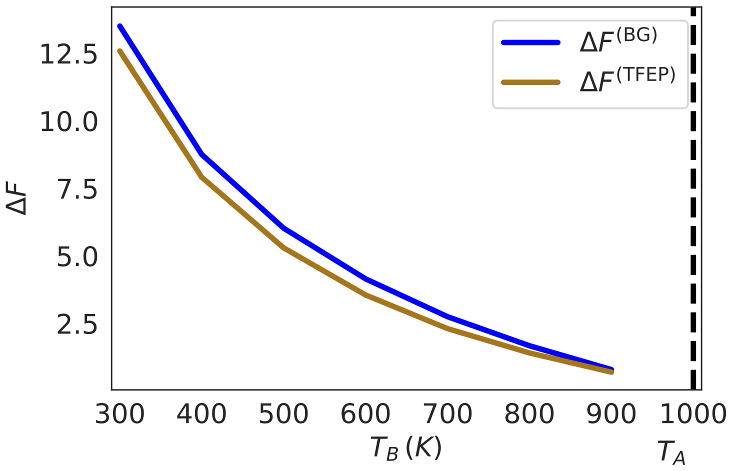


Figure S2: A comparison of the two free energy estimators, evaluated using the latent TI model. As can be seen in the figure, the derived bound from Equation (11) holds for all target temperatures $T_B$.

ated. To address the issue, a number of the outlier values were filtered out, hopefully leading to more reliable and numerically stable estimates.

The filtering was done by calculating the interquartile range (IQR), which in our case is defined as the difference between the lower and upper quartiles of the $w^{l/a}/\psi/\phi$-distributions. An outlier was identified as a data point lying outside the range $[Q_1 - k \cdot \mathrm{IQR}, Q_3 + k \cdot \mathrm{IQR}]$, where $Q_1$ and $Q_3$ represent the lower and upper quartiles respectively, and $k$ is a constant set to a large value. The constant $k$ was set to $k = 10$ in the lower-dimensional case and to $k = 100$ in the higher-dimensional case. Note that removing too many samples will reduce sampling efficiency, since samples are filtered out, resulting in a lower total amount of samples in the end. It is generally also not a good approach to remove higher valued statistical weights, as they theoretically correspond to more relevant samples. Setting $k = 1.5$ corresponds to $3\sigma$ if the data is normally distributed, so we anticipate that our choice only removes the most extreme outliers.

## ADW Dataset Generation

We generate a dataset of samples drawn from the Boltzmann distribution corresponding to a one-dimensional asymmetric double well system, defined through the energy

$$E(x) = 4(x^2 - 1)^2 + 0.5x. \tag{12}$$

We sample at inverse temperatures, $(kT)^{-1} \in \{0.25, 0.5, 0.75, 1.0, 1.25, 1.5, 1.75, 2.0\}$, by integrating the overdamped Langevin dynamics and obtain the dataset illustrated in Figure S3.

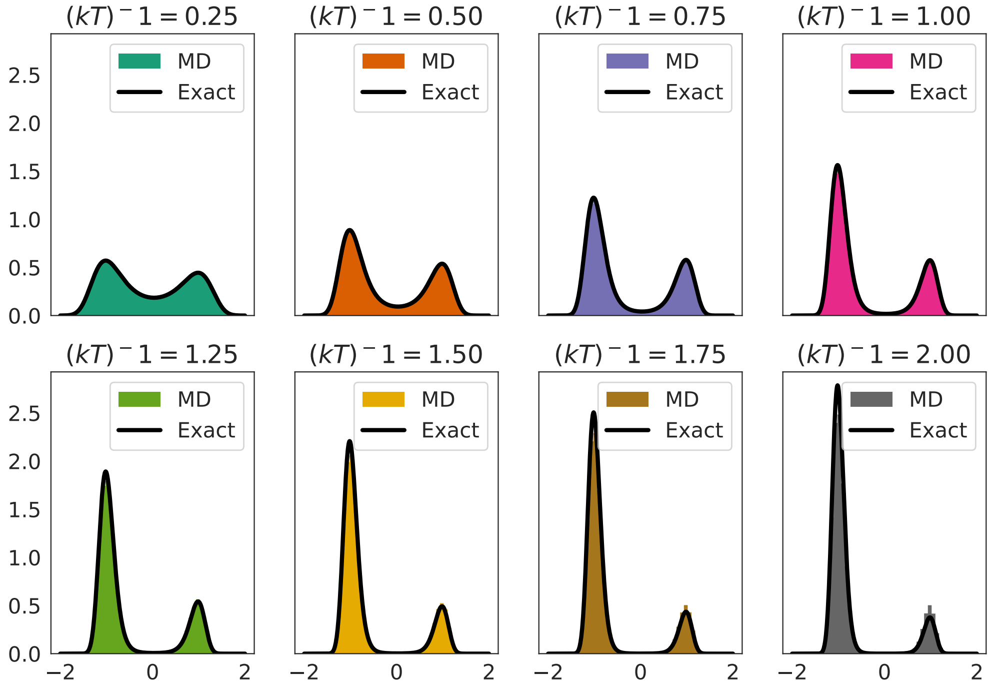


Figure S3: An illustration of the sampled ADW dataset. We obtain samples by integrating the overdamped Langevin dynamics at inverse temperatures $(kT)^{-1} \in \{0.25, 0.5, 0.75, 1.0, 1.25, 1.5, 1.75, 2.0\}$, with friction coefficient $\gamma = 1.0$ and with a discretized time step $\Delta t = 0.001$.

## Hyperparameters for ADW System

When training our ambient TI models on the one-dimensional ADW system we use the parameters in Table A1.

Table A1: Hyperparameters for ambient TI models trained on the one-dimensional ADW model system.

| Parameter | Value |
|---|---|
| Batch Size | 512 |
| Hidden Layer Size | 256 |
| Number of MLP Layers | 5 |
| Learning Rate | $10^{-4}$ |
| Weight Decay | $10^{-5}$ |
| Number of Epochs | 100 |
| $\gamma(t)$ | $\sqrt{0.9t(1-t)}$ |
| NODE Solver | Dopri5 |
| Relative Error Tolerance | $10^{-4}$ |
| Absolute Error Tolerance | $10^{-4}$ |

## Hyperparameters for Molecular Systems

When training our molecular ambient TI models we use the parameters in Table A2. For latent TI, we use the parameters in Table A3.

Table A2: Hyperparameters for ambient TI models trained on the QM9 molecular systems.

| Parameter | Value (Big molecule/Small molecule) |
|---|---|
| Batch Size | 128/256 |
| Hidden Layer Size | 256/128 |
| Number of Message/Update Blocks | 5/5 |
| Learning Rate | $10^{-4}/10^{-4}$ |
| Weight Decay | 0/0 |
| Number of Epochs | 150/150 |
| $l_0$ | 100/100 |
| $\gamma(t)$ | $\sin^2(\pi t)/\sin^2(\pi t)$ |
| NODE Solver | Dopri5 |
| Relative Error Tolerance | $10^{-5}$ |
| Absolute Error Tolerance | $10^{-5}$ |

Table A3: Hyperparameters for latent TI models trained on the QM9 molecular system.

| Parameter | Value |
|---|---|
| Batch Size | 256 |
| Hidden Layer Size | 128 |
| Number of Message/Update Blocks | 5 |
| Learning Rate | $10^{-4}$ |
| Weight Decay | 0 |
| Number of Epochs | 110 |
| $l_0$ | 75 |
| $\gamma(t)$ | 0 |
| NODE Solver | Dopri5 |
| Relative Error Tolerance | $10^{-5}$ |
| Absolute Error Tolerance | $10^{-5}$ |

## Single vs. Multi-Temperature Training

As shown in Figure S4, training the model with only a single temperature leads to problems, specifically the appearance of incorrect states in the torsion angles. However, when we train the model with multiple temperatures, we see a noticeable improvement. This multi-temperature training helps eliminate these incorrect states, resulting in more accurate torsion angle predictions for each single temperature.

## gEDMD Model Selection

An important decision when applying the gEDMD approach is selecting the dictionary, for example, the set of basis functions $\phi(\mathbf{x})$. Kernel-based methods offer effective approximations, often yielding accurate results. These kernel functions can be further approximated using random Fourier features (RFF).[4,5] The general procedure involves choosing the number of Fourier features, $p$, and the kernel bandwidth, $\sigma$, based on the VAMP score.[6]

We present the model selection results for the molecular systems in Figure S5, where we run gEDMD on a single torsion angle for the N-Me and on the six of 21 torsion angles for the 3p2y1 molecule showing multimodal distributions (Torsion index {2, 5, 8, 11, 15, 18} as shown in Fig. S1).

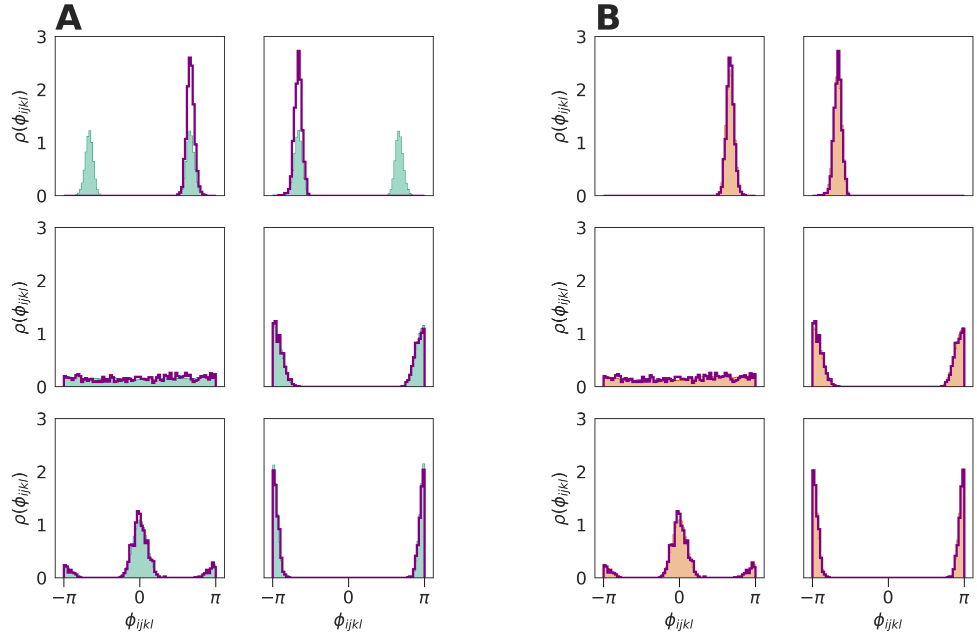


Figure S4: Comparison of torsion angles $\phi_{ijkl}$ in the N-Me, sampled from latent TI model when trained on a single temperature versus multiple temperatures. Transformations are made from standard normally distributed noise into molecular conformations at target temperature $T_A$ = 800K. **A** Latent TI model trained on data at a single temperature $T_{\text{train}}$ = 800K compared to MD simulated reference values. **B** Latent TI model trained on data at multiple temperatures $T_{\text{train}} = \{300, 400, 500, 600, 700, 800, 900, 1000\text{K}\}$, compared to MD simulated reference values.

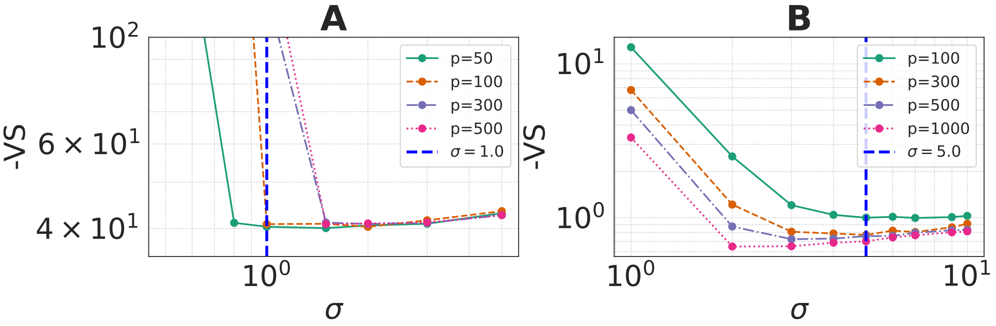


Figure S5: VAMP scores as a function of the kernel bandwidth $\sigma$ for different numbers of Fourier features $p$. **A** We select $p = 50$ and $\sigma = 1.00$ as the model parameters for N-Me system. **B** We select $p = 300$ and $\sigma = 5.00$ as the model parameters for 3p2y1y system.

## MSM For Different Systems

We present the eigenvalues estimated using Markov state models (MSMs)[7] for a 1D double-well system, which correspond to the metastable states. Our results show a difference in the

transition rates between underdamped and overdamped simulations, as shown in Figure S6.

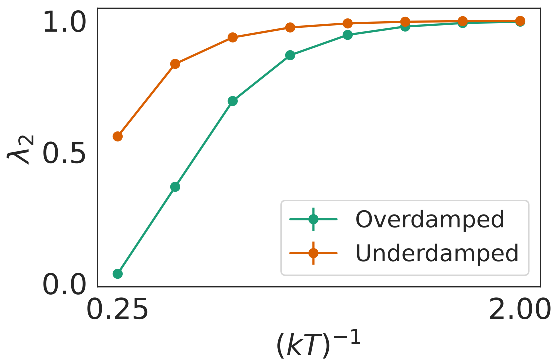


Figure S6: A comparison of transition rates for underdamped and overdamped simulations, evaluated using the MSM model.

# References

(1) Molgedey, L.; Schuster, H. G. Separation of a mixture of independent signals using time delayed correlations. *Phys. Rev. Lett.* **1994**, *72*, 3634–3637.

(2) Noé, F.; Olsson, S.; Köhler, J.; Wu, H. Boltzmann generators: Sampling equilibrium states of many-body systems with deep learning. *Science* **2019**, *365*, eaaw1147.

(3) Jarzynski, C. Targeted free energy perturbation. *Phys. Rev. E* **2002**, *65*, 046122.

(4) Rahimi, A.; Recht, B. Random features for large-scale kernel machines. *Advances in neural information processing systems* **2007**, *20*.

(5) Nüske, F.; Klus, S. Efficient approximation of molecular kinetics using random Fourier features. *The Journal of Chemical Physics* **2023**, *159*.

(6) Wu, H.; Noé, F. Variational approach for learning Markov processes from time series data. *Journal of Nonlinear Science* **2020**, *30*, 23–66.

(7) Prinz, J.-H.; Wu, H.; Sarich, M.; Keller, B.; Senne, M.; Held, M.; Chodera, J. D.; Schütte, C.; Noé, F. Markov models of molecular kinetics: Generation and validation. *The Journal of chemical physics* **2011**, *134*.
